# Gut microbiota promote the inflammatory response in the pathogenesis of systemic lupus erythematosus

**DOI:** 10.1186/s10020-019-0102-5

**Published:** 2019-08-01

**Authors:** Yiyangzi Ma, Xiaoxue Xu, Mengtao Li, Jun Cai, Qiang Wei, Haitao Niu

**Affiliations:** 1 NHC Key Laboratory of Human Disease Comparative Medicine, The Institute of Laboratory Animal Sciences, Chinese Academy of Medical Sciences & Peking Union Medical College; Key Laboratory of Human Diseases Animal Model, State Administration of Traditional Chinese Medicine, Beijing, China; 2 Department of Core Facility Center, Capital Medical University, Beijing, China; 3 Division of Rheumatology, Peking Union Medical College Hospital, Chinese Academy of Medical Sciences & Peking Union Medical College, Beijing, China; 4 Hypertension Center, Fuwai hospital, State Key Laboratory of Cardiovascular Disease of China, National Center for Cardiovascular Disease of China, Chinese Academy of Medical Sciences & Peking Union Medical College, Beijing, China

**Keywords:** Gut microbiota_1_, Fecal microbiota transplantation_2_, Systemic lupus erythematosus_3_, Immune response_4_, Lupus susceptibility gene_5_

## Abstract

**Objectives:**

Systemic lupus erythematosus (SLE) is a chronic autoimmune disease whose onset and progression are affected by genetic and environmental factors. The purpose of this study is to identify the influence of gut microbiota in the pathogenesis of SLE, and to investigate the mechanism involved.

**Methods:**

Fecal microbiota from C57/BL6 mice and SLE prone mice were examined using next-generation sequencing (NGS). Germ free mice were given fecal microbiota transplantation (FMT), and their gut microbiome and gene expression in recipients’ colons were examined by NGS. The anti-double stranded DNA (anti-dsDNA) antibodies in recipients were determined using an enzyme-linked immunosorbent assay (ELISA). The immune cell profiles of mice were analyzed by flow cytometry at the 3rd week after FMT, and the expression of genes associated with SLE after FMT was determined using quantitative real-time PCR (qRT-PCR).

**Results:**

The fecal microbiota of SLE mice had lower community richness and diversity than healthy mice. Fecal microbiota of recipient mice were similar to their donors. Fecal microbiome from SLE mice could lead to a significant increase of anti-dsDNA antibodies and promote the immune response in recipient mice. Our results also indicated that fecal microbiome from SLE mice resulted in significant changes in the distribution of immune cells and upregulated expression of certain lupus susceptibility genes.

**Conclusions:**

SLE is associated with alterations of gut microbiota. Fecal microbiome from SLE mice can induce the production of anti-dsDNA antibodies in germ free mice and stimulate the inflammatory response, and alter the expression of SLE susceptibility genes in these mice.

**Electronic supplementary material:**

The online version of this article (10.1186/s10020-019-0102-5) contains supplementary material, which is available to authorized users.

## Introduction

Systemic lupus erythematosus (SLE) is a chronic autoimmune disease that affects connective tissues and is characterized by the presence of serum anti double-stranded DNA (Anti-dsDNA) antibodies (Rahman and Isenberg 2008). An increased titer of anti-dsDNA antibodies is generally regarded as a specific diagnostic marker for human SLE (Mummert et al. 2018), although not all patients have high titers of anti-dsDNA antibodies (Sinico et al. 2002). In lupus-prone mice, a high titer of anti-dsDNA antibodies is also a specific marker for lupus, and some studies showed that a high titer of anti-dsDNA antibodies also correlated with the severity of nephritis (Shen et al. 1998; Blatt and Glick 1999; Niu et al. 2008; Niu et al. 2013). The exact etiology and pathogenic mechanism of SLE remains elusive, but genetic and environmental factors seem to play important roles (Picascia et al. 2015). Multiple susceptibility genes and loci are associated with SLE (Deng and Tsao 2017), but genetic factors alone do not cause SLE, because certain environmental and other factors also contribute to this disease. According to the “Hygiene Hypothesis”, environmental pressure, including microorganisms, can affect the body’s immune system (Okada et al. 2010). The recent development of NGS also provides a useful tool for microbiome research (Reuter et al. 2015). Many studies demonstrated that alterations of gut microbiota correlate with a variety of diseases, including autoimmune disorders such as SLE (Ma et al. 2015; Liu et al. 2019; Shi et al. 2019).

A mouse model of SLE, B6.NZM-Sle1^NZM2410/Aeg^Sle2^NZM2410/Aeg^Sle3^NZM2410/Aeg^/LmoJ congenic mouse (TC), contains three NZM2410-derived SLE susceptibility loci that are coexpressed in C57/B6 (B6) background (Morel et al. 2000). These mice produce anti-dsDNA IgG, and the titer of these antibodies correlate with disease activity (Mohan et al. 1998). Additional research has confirmed that these mice also have increased activation of B cells and T cells, as in patients with SLE (Morel et al. 2000; Sobel et al. 2002).

Gut microbiota inhabit the intestinal tract, and play vital roles in metabolic functions, maintenance of the intestinal barrier, and immune system function (Backhed et al. 2005; Ley et al. 2006). Dysbiosis of gut microbiota correlates with many diseases, including autoimmune diseases such as inflammatory bowel disease (IBD) and rheumatoid arthritis (RA) (Shamriz et al. 2016). Several recent studies reported the role of intestinal microbiota in the pathogenesis of SLE. Hevia et al. described the dysbiosis of gut microbiota in SLE patients (Hevia et al. 2014) and Zhang et al. described composition of gut microbiota in a murine model of SLE that was different from the control (Zhang et al. 2014). Subsequent studies demonstrated that alterations in the gut microbiome were related to lupus (Mu et al. 2017a; Mu et al. 2017b; Luo et al. 2018). Although these previous studies showed SLE is possibly associated with a change of gut microbiota, much remains unknown about the role of gut microbiota in the pathogenesis of SLE and the underlying mechanism.

The objective of this study is to identify whether the gut microbiota of lupus-prone mice can induce lupus-like symptoms, the influence of gut microbiota on the pathogenesis of lupus, and the possible underlying mechanism. The present study characterized the gut microbiota of healthy mice, mice with lupus, and germ-free (GF) mice that transplanted with fecal microbiota from SLE mice or C57/B6 mice. We measured serum levels of anti-dsDNA antibodies, and used flow cytometry to analyze the distributions of immune cells in the different groups of mice. We also used transcriptome sequencing for gene expression functional analysis, and qRT-PCR to confirm the expression of known SLE susceptibility genes of FMT recipients.

## Materials and methods

### Mice

C57BL/6 mice (B6 mice) and B6.NZM-Sle1^NZM2410/Aeg^Sle2^NZM2410/Aeg^Sle3^NZM2410/Aeg^/LmoJ mice, called TC (SLE), were purchased from The Jackson Laboratory (Bar Harbor, ME, USA), and were bred and maintained in specific pathogen-free (SPF) in the Institute of Laboratory Animal Sciences (ILAS) at the Chinese Academy of Medical Sciences & Peking Union Medical College. C57/B6J germ free mice (GF mice) were generated and provided by ILAS, which is a member of (and accredited by) the American Association for the Accreditation of Laboratory Animal Care. All experiments were performed in accordance with the guidelines of the Institutional Animal Care and Use Committees of the ILAS. All mice used for experiment were female. TC mice (female, 34–36 weeks) were used after development of high titers of anti-dsDNA autoantibodies. C57/B6 mice were sex and age matched. All GF mice were used after the age of 8 weeks and were housed independently, one mouse per cage and one group per isolator.

### Fecal microbiota collection and transplantation

Feces from 11 control mice (C57/B6) and 11 TC (SLE) mice, were separately collected. 1 g of each mixed sample was suspended in 5 mL of phosphate-buffered saline solution (PBS), vortexed thoroughly, and administered as a 200 μL aliquot. Adult GF mice were divided into 3 groups (5 mice per group): a GF + PBS group was gavaged with sterile PBS; the GF + B6 group was gavaged with feces from B6 mice; and the GF + SLE group was gavaged with feces from TC (SLE) mice. Fecal gavage was performed 5 times, once every other day for 10 days. All mice were sacrificed at week-3 after the final gavage.

### Microbial DNA extraction and sequencing

The feces of C57/B6 mice and TC (SLE) mice were collected in sterile tubes and quickly frozen at − 80 °C. The feces of recipient GF mice were collected at the 3rd week after FMT. Total genome DNA was extracted using the CTAB/SDS method, and DNA concentration and purity were monitored on 1% agarose gels. DNA was diluted to 1 ng/μL in sterile water. PCR amplification of the V4 regions of bacterial 16S rRNA genes for paired end sequencing (2 × 250 bps) on the IonS5™XL platform was performed using universal primer sequences with the barcode (515F, 5′-GTGCCAGCMGCCGCGGTAA-3′; 806R, 5’GGACTACHVGGGTWTCTAAT-3′). The library was sequenced on an IonS5™XL platform, and 400 /600 bp single-end reads were generated. Sequence analysis was performed using Uparse software (ver. 7.0.1001) (Edgar 2013). Sequences with 97% or more similarity were assigned to the same operational taxonomic unit (OTU). For each representative sequence, the Silva Database (https://www.arb-silva.de/) was used based on Mothur algorithm to annotate taxonomic information. The abundance of each OTU was normalized relative to the sample with the fewest sequences. Alpha diversity and Beta diversity indices were calculated using QIIME (ver. 1.7.0), and results are displayed using R software (ver. 2.15.3). LEfSe analysis is conducted by LEfSe software (the default LDA score is 4) (Segata et al. 2011). The differential abundance of OTUs was filtered by a false discovery rate-adjusted significance of 0.05 or less using linear discriminant analysis (LDA). Average robust fold-change for each OTU was computed using the fcros package in R software by pairwise sample comparison with the default quartile feature exclusion criteria, based on fold-change rank-order statistics (Dembele and Kastner 2014). The SourceTracker analysis was conducted in R using default parameter settings, in which different categorical probabilities were used for calling a certain ratio of source present. For each sample, predicted proportion for each of 6 potential sources ((i.e., Bedding, Food, Water, Feces from C57/B6 mice (B6), Feces from TC mice (TC) and Unknown). Consistency between the predicted source and original ecotype was used to calculate general SourceTracker prediction accuracy.

### RNA extraction and transcriptome sequencing

The colon tissues of GF mice were collected and stored at − 80° immediately after euthanization. Total RNA was isolated using the TRIzol reagent (Invitrogen, CA, USA), and 3 μg RNA per sample was used for analysis. Sequencing libraries were generated using NEBNext® UltraTM RNA Library Prep Kit for Illumina® (New England Biolabs, USA) following manufacturer’s recommendations, and index codes were added for attribution of each sample. The library was sequenced on an Illumina Hiseq platform, and 125/150 bp paired-end reads were generated. Raw data (raw reads) in the fastq format were first processed through in-house Perl scripts. Clean data (clean reads) were then obtained by removing reads containing adapter sequences, poly-N sequences, and low quality reads from the raw data. Reference sequences were from STAR, and paired-end clean reads were aligned to the reference genome using STAR (ver. 2.5.1b). HTSeq (ver. 0.6.0) was used to count the read numbers for each gene. The number of fragments per kilobase of transcript per million mapped reads of each gene was calculated based on the length of the gene and the number of reads mapped to that gene. Differential expression genes in the two groups were determined by using the DESeq2 in R software. The Cluster Profiler in R software was used to test the statistical significance of enrichment of different genes, based on pathways in the Kyoto Encyclopedia of Genes and Genomes. Gene Ontology (GO) enrichment analysis of differentially expressed genes was implemented using the cluster Profiler in R software, with correction for gene length bias. The differential expression of genes was analyzed by multiple hypothesis testing, based on the principle of negative binomial distribution. A significant difference is presented as a false-discovery rate (FDR) adjusted *p*-value, is also called a q-value.

### Quantitative reverse transcription PCR (qRT-PCR)

RNA was extracted using the TRIzol Reagent (Invitrogen, CA, USA), and cDNA synthesis was performed using the Transcriptor First Strand cDNA Synthesis Kit (Roche, Germany). Then, qRT-PCR was performed using the Applied Biosystem StepOne Plus PCR system with TB Green™ Premix Ex Taq™ II (TaKaRa Bio., Japan). The expression of *IRF7* and *CSK* genes were determined relative to GAPDH using appropriate primers (Table 1).

**Table 1:** Primers used for qRT-PCR

Gene	Primer
IRF7	Forward: GCGTACCCTGGAAGCATTTC
	Reverse: GCACAGCGGAAGTTGGTCT
CSK	Forward: TCATGTGGGGTCTAGCAGGTA
	Reverse: AGGGCTTGGCTATCTGACTG
GAPDH	Forward: AACTTTGGCATTGTGGAAGG
	Reverse: GGATGCAGGGATGATGTTCT

### Enzyme-linked immunosorbent assay (ELISA) for serum IgG anti-dsDNA antibodies

Serum IgG anti-dsDNA antibodies were measured using ELISA as previously described (Niu et al. 2008; Niu et al. 2013). Serum samples were detected by alkaline phosphatase conjugated goat anti-mouse IgG and developed by p-nitrophenyl phosphate PNPP (Sigma, USA), and the absorbance at 405 nm was determined using an ELISA reader (Omega, Germany).

### Isolation of splenocytes and lamina propria lymphocytes (LPLs)

Spleens were removed and cut into pieces in cold PBS. After filtration through a 200-gauge steel mesh and removal of red blood cells with an RBC lysis buffer (BD Biosciences, USA), splenocytes were collected for further experimentation. Preparation of LPLs was performed as previously described (Weigmann et al. 2007).

### Flow cytometry

Splenocytes and LPLs were incubated with rat anti-mouse CD16/CD32 mAb (clone 2.4G2, BioLegend, USA) for 30 min to block Fc receptors. Cells were then stained on ice with fluorochrome-conjugated monoclonal antibodies for 30 min. Intracellular staining were treated with fixation and permeabilization kit (BD Pharmingen™, USA), cells were then stained with appropriate antibodies. Antibodies used for experiment were listed in Table 2. Cells were acquired on the Symphony A5 cytometer (BD Biosciences). FlowJo software (ver. 10.5.3, BD Biosciences) was used for analysis of flow cytometry data.

**Table 2:** Antibodies used for flow cytometry experiments. All antibodies were from BD Bioscience

Marker	Fluorochrome
CD19	BUV737
B220 (CD45R)	BUV395
CD138	BV421
CD38	PE
CD95	BV510
IgM	APC
IgD	BV786
PNA	FITC
CD93	BV650
CD3e	APC-Cy7
CD4	BUV395
CD8a	Percp-Cy5.5
CD25	BB515
Foxp3	BV421
RORγt	BV786
Live/Death	FVS700

### Statistical analysis

GraphPad Prism software (San Diego, CA) was used for statistical analysis. A Mann-Whitney *t*-test was used to compare two groups, and a one-way ANOVA was used to compare multiple groups. A *P* value below 0.05 was considered significant.

## Results

### SLE mice have reduced richness and abundance of fecal microbiota

**Fig. 1:**
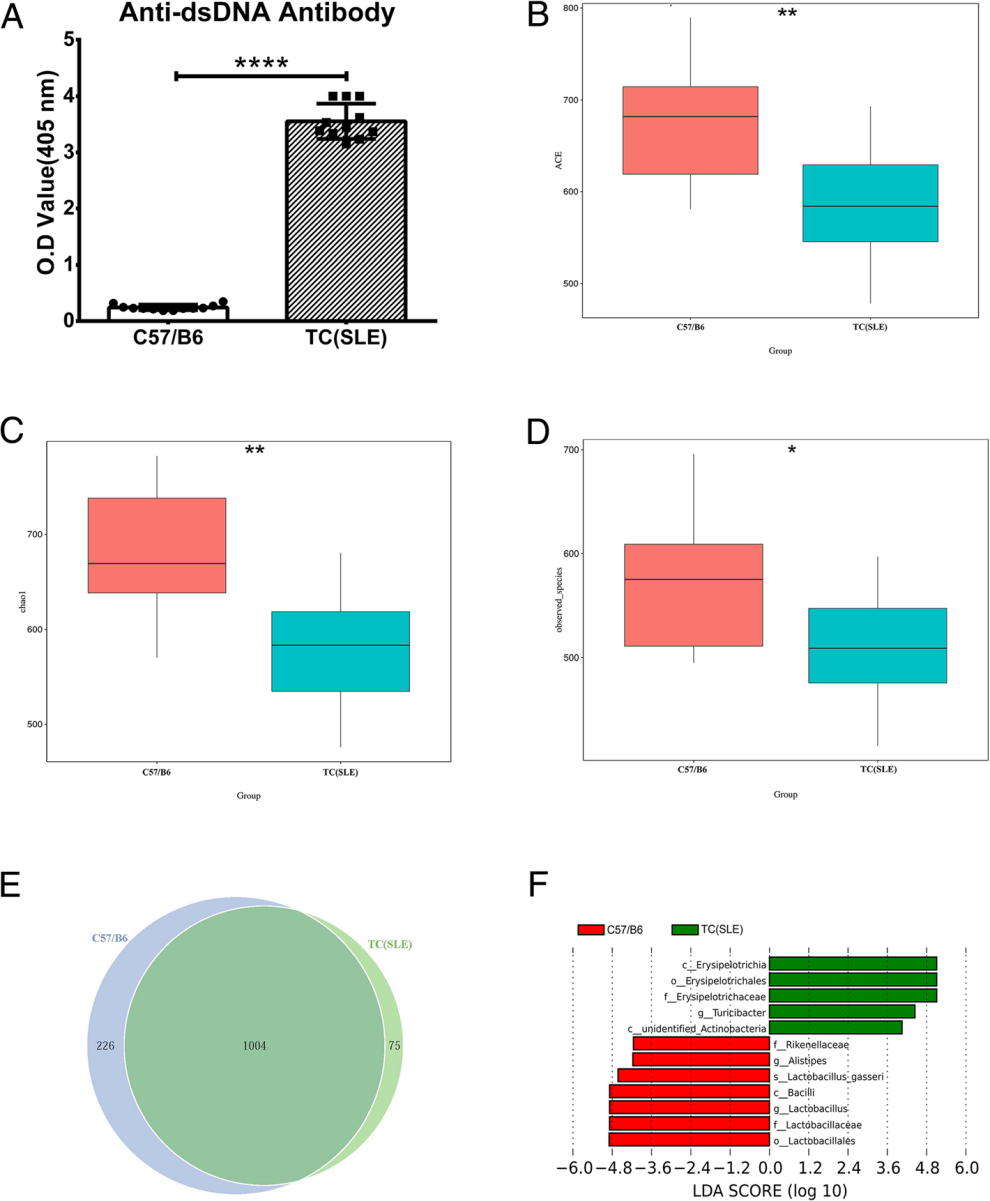
Establishment of the diseased SLE model and analysis of gut microbiota in SLE mice and C57/B6 mice. (A) Anti-dsDNA antibody titers in TC (SLE) mice and C57/B6 mice (11 mice per group). (B-F) Feces of each group were collected for analysis of bacterial 16S DNA from the colorectum and determination of the (B) ACE index and (C) Chao1 index, *P < 0.05, **P < 0.01. (D) Comparison of observed species comparison between groups. *P < 0.05. (E) Unique and shared microbiota of the two groups (Venn diagram). (F) LDA scores for different bacterial taxa of the two groups (p, phylum; c, class; o, order; f, family; g, genus)

We first assessed the establishment of diseased model of SLE by measuring serum anti-dsDNA antibody titers, a defining characteristic of SLE. The results show that TC (SLE) mice had significantly greater levels of anti-dsDNA antibodies than C57/B6 mice (Fig. 1a).

We determined the diversity of fecal microbiota by calculation of alpha diversity indices. The results showed that C57/B6 mice had greater community richness than SLE mice based on the ACE index and the Chao1 index (Fig. 1b, c). The C57/B6 mice also had a significantly higher community diversity based on observed species indices (Fig. 1d). The Venn diagram reflected distribution of OTU clusters between C57/B6 and TC (SLE) groups. A Venn diagram of the OTU clusters in the C57/B6 and TC (SLE) groups indicates that 1004 OTUs were shared, 226 OTUs were specific to C57/B6 mice, and only 75 OTUs were specific to TC (SLE) mice (Fig. 1e).

We also determined the beta diversity of feces from different mice using the LDA score, which identifies features consistent with biologically meaningful categories. An LDA score above 4 indicates the greatest difference in taxa from the phylum to the species level. Thus, 5 taxa were enriched in TC (SLE) mice, and 7 were enriched in C57/B6 mice (Fig. 1f). The TC (SLE) microbial biomarkers were in the *Firmicutes* phylum (*Erysipelotrichia, Erysipelotrichales, Erysipelotrichaceae*, and *Turicibacter*) and the *Actinobacteria* phylum (unidentified *Actinobacteria*); the C57/B6 microbial biomarkers were in the *Bacteroidetes* phylum (*Rikenellaceae, Alistipes*) and the *Firmicutes* phylum (*Lactobacillus_gasseri, Bacilli, Lactobacillus, Lactobacillaceae, Lactobacillales*). These data clearly indicate a decreased gut microbiome richness in mice with SLE and suggest that certain microbiological changes may be associated with the pathogenesis of SLE.

### Fecal microbiota from SLE mice alter gut microbiota and induce production of auto-antibodies against dsDNA in GF mice

**Fig. 2:**
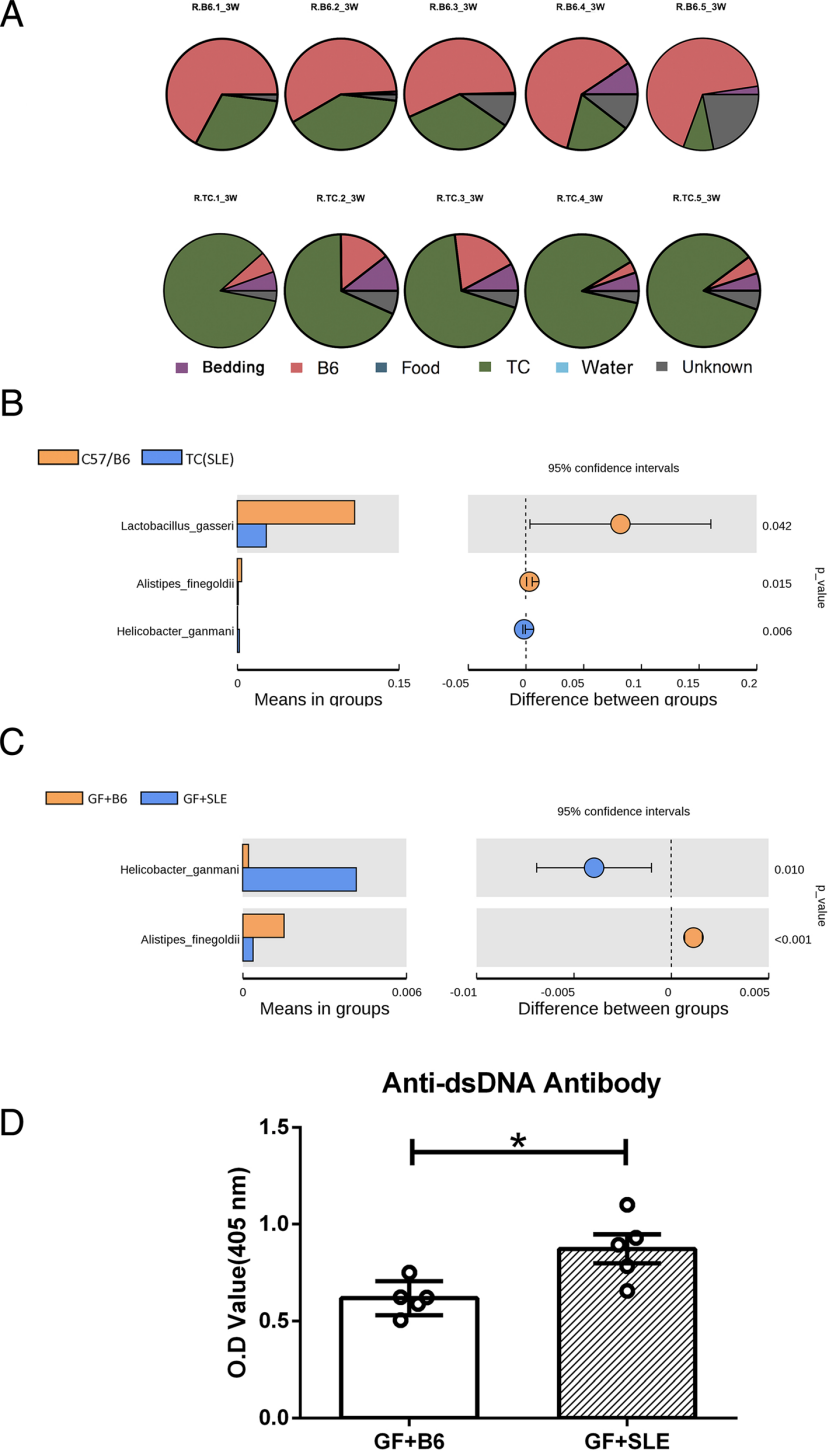
Effect of FMT from control mice and SLE mice into GF mice. (A) SourceTracker results of recipient mice (5 mice per group). The first row shows the result of each mouse in the GF + B6 group and the second row shows the result of each mouse in the GF + SLE group. (B) Significantly different species in the SLE group and C57/B6 group (11 mice per group; left); difference in mean proportions between the groups, *P*-values, and 95% confidence intervals (right). 16 s rRNA gene sequences were assigned to the species level based on greater than 97% identity to reference sequences (see Materials and methods). (C) Significantly different species in the GF + SLE group and the GF + B6 group (5 mice per group); difference in mean proportions between the groups, P-values, and 95% confidence intervals (right). (D)Anti-dsDNA antibody titers of mice in the GF + SLE group and the GF + B6 group (5 mice per group). Data represent one of three independent experiments, and error bars represent means ± SEMs

We next examined the impact of fecal microbiota from SLE mice and control mice separately transplanted into germ free mice. By using SourceTracker analysis we determined the contributions of fecal microbiota transplantation from TC (SLE) mice and C57/B6 mice to GF mice. The results (Fig. 2a, Table 3) indicated that recipients of C57/B6 feces (Group GF + B6) had 61.9% (±4.5) of the microbiota from C57/B6 mice, and that recipients of TC (SLE) feces (Group GF + SLE) had 78.8% (±8.7) of the microbiota from TC mice. For this analysis, we collected all fecal pellets at the 3rd week after FMT. The alpha diversity indices of donor mice and recipient mice were listed in Additional file 1: Table S1. The community composition of microbiota and Principal Component Analysis (PCA) of the microbiota in donor mice and recipient mice were shown in Additional file 2: Figure S1 and S2. These supplementary data indicated that the microbiota in recipient mice was different between groups with different feces source, and maintain the same tendency of diversity as donors. Figure 2b showed the significant differences of microbiome species in C57/B6 mice and TC (SLE) mice. Figure 2c showed significant differences of microbiome species as of the recipient mice in GF + B6 group and GF + SLE group. In particular, *Alistipes_finegoldii* and *Helicobactor_ganmani* maintained the same trends in recipient mice (Fig. 2b, c). We detected total IgG antibodies in serum but there were no significant differences in GF + B6 group as compared in GF + SLE group (Additional file 3: Figure S3) 3 weeks after FMT. Strikingly, mice that received SLE feces had higher antibody titers against dsDNA than mice that received B6 feces (Fig. 2d). These results indicate that GF mice which received SLE fecal developed higher anti-dsDNA antibodies.

**Table 3:** SourceTracker results for mice in the GF + B6 group and the GF + SLE group

Sink		Source (%)					
Group	Sample	Bedding	B6	Food	TC	Water	Unknown
GF + B6	R.B6.1	0.02	67.13	0	30.94	0	1.91
	R.B6.2	0.81	57.55	0	39.81	0	1.83
	R.B6.3	0.39	56.39	0	33.61	0	9.61
	R.B6.4	9.34	61.47	0.01	18.68	0	0.5
	R.B6.5	2.39	66.99	0.01	8.76	0	21.85
GF + SLE	R.TC.1	5.42	6.09	0	85.53	0	2.96
	R.TC.2	0.54	14.6	0	68.15	0	6.71
	R.TC.3	7.85	19.05	0	68.2	0	4.9
	R.TC.4	5.32	3.3	0	87.95	0	3.43
	R.TC.5	5.06	5.15	0	84.34	0	5.45

### Fecal microbiota from SLE mice increase intestinal mucosal immune response in GF mice

**Fig. 3:**
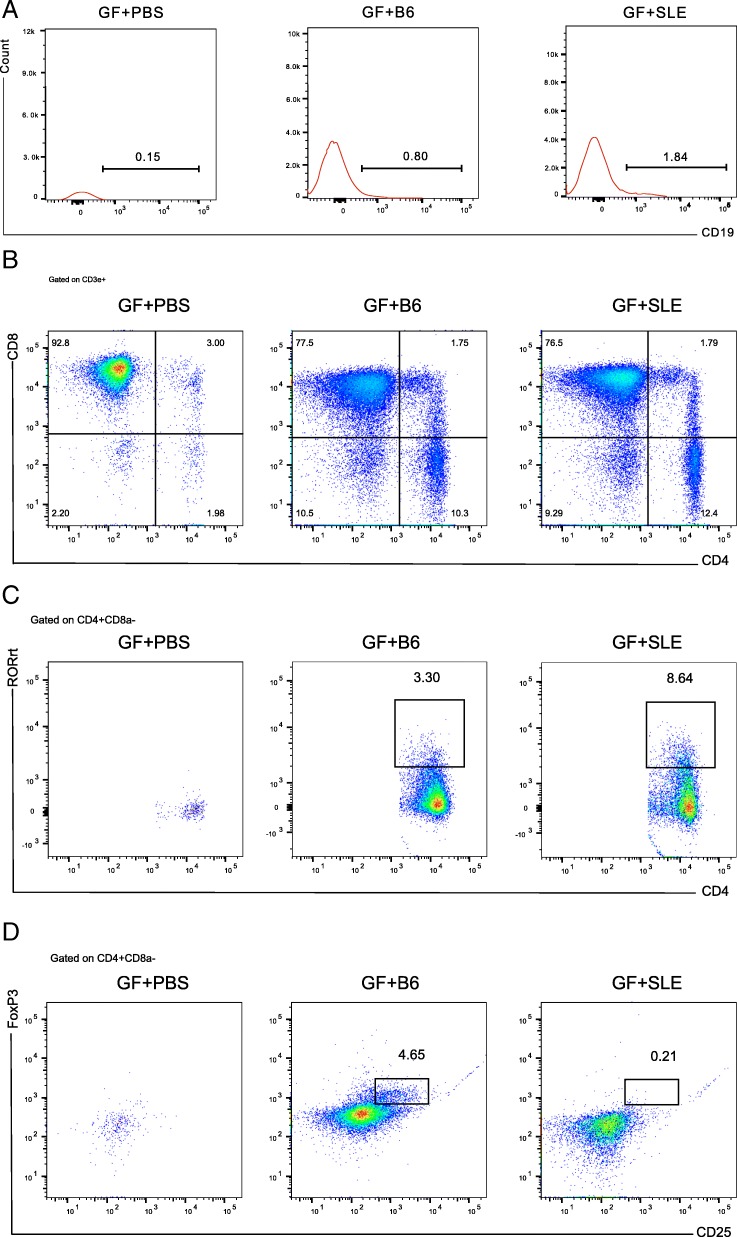
Flow cytometry analysis of the distribution of lymphocytes in the lamina propria of GF + PBS mice, GF + B6 mice and GF + SLE mice. (**a**) Histograms of CD19^+^ B cells. The number is the percentage of CD19^+^ B cells of live cells. The red histograms show CD19^+^ cells of live cells. The right statistical results show percentages of B cells in groups (5 mice per group). (**b**) Plots represent CD4^+^ CD8a^−^ T cells (left) and the percentages of CD4^+^ CD8a^−^ T cells in 3 groups (5 mice per group; right). (**c**) Plots represent CD4^+^ RORγt ^+^ T cells (left) and percentages of CD4^+^ RORγt ^+^ cells in 3 groups (5 mice per group; right). (**d**) Plots represent regulatory T cells (Tregs; left) and percentages of Treg cells in 3 groups (5 mice per group; right). Data represent one of three independent experiments. A Mann-Whitney *t*-test was used to compare groups, and error bars represent means ± SEMs. ^*^P < 0.05, ^**^P < 0.01, ^***^P < 0.001, ^****^P < 0.0001

The microbiota introduced by FMT can contact and act on intestinal mucosa, and stimulate immune response in the lamina propria (LP) (Montalban-Arques et al. 2018). B cells and T cells constitute the part of mucosal immunity. Thus, we examined the lymphocyte distributions in the LP of mice receiving different treatments. Analysis of B cells and its subsets in GF + PBS mice indicated very few B cells (CD19^+^), and no detectable B cell subsets due to no microbiota stimulation. Mice in the GF + SLE and GF + B6 groups had more B cells and B cell subsets. Compared with B6 feces, SLE feces had a greater effect to stimulate B cells (Fig. 3a).

Analysis of T cells and subsets indicated that CD4^+^ T cells (CD3e^+^CD4^+^CD8^−^) and its subsets in GF + PBS mice were insufficient for quantification due to no microbiota stimulation. Mice in the GF + SLE group and GF + B6 groups had increased numbers of CD4^+^ T cells as compared with GF + PBS group. However, there were no significant differences in GF + B6 group and GF + SLE group (Fig. 3b). Due to the insufficient number of CD4^+^ T cells subsets in GF + PBS group, no comparisons of this group with other groups were performed. To analyze T cell subsets, we gated regulatory T cells (Treg, CD3e^+^CD4^+^CD8a^−^CD25^+^Foxp3^+^), CD4^+^RORγt^+^ T cells (CD3e^+^CD4^+^CD8a^−^RORγt^+^), and Th17 cells (CD3e^+^CD4^+^ CD8a^−^IL17a^+^). Although there were no significant differences in the distributions of Th17 cells between groups (data not shown), mice in the GF + SLE group had significantly increased CD4^+^ RORγt^+^ T cells (Fig. 3c). These cells express RORγt, a master transcriptional factor that can direct the differentiation of Th17 cells (Bassolas-Molina et al. 2018). Treg cells were significantly less abundant in the GF + SLE group than in the GF + B6 group (Fig. 3d). These data demonstrate that FMT induced a intestinal mucosal immune response, and that SLE feces elicited a more intense response than B6 feces.

### Fecal microbiota from SLE mice induce stronger splenic immune response in GF mice

**Fig. 4:**
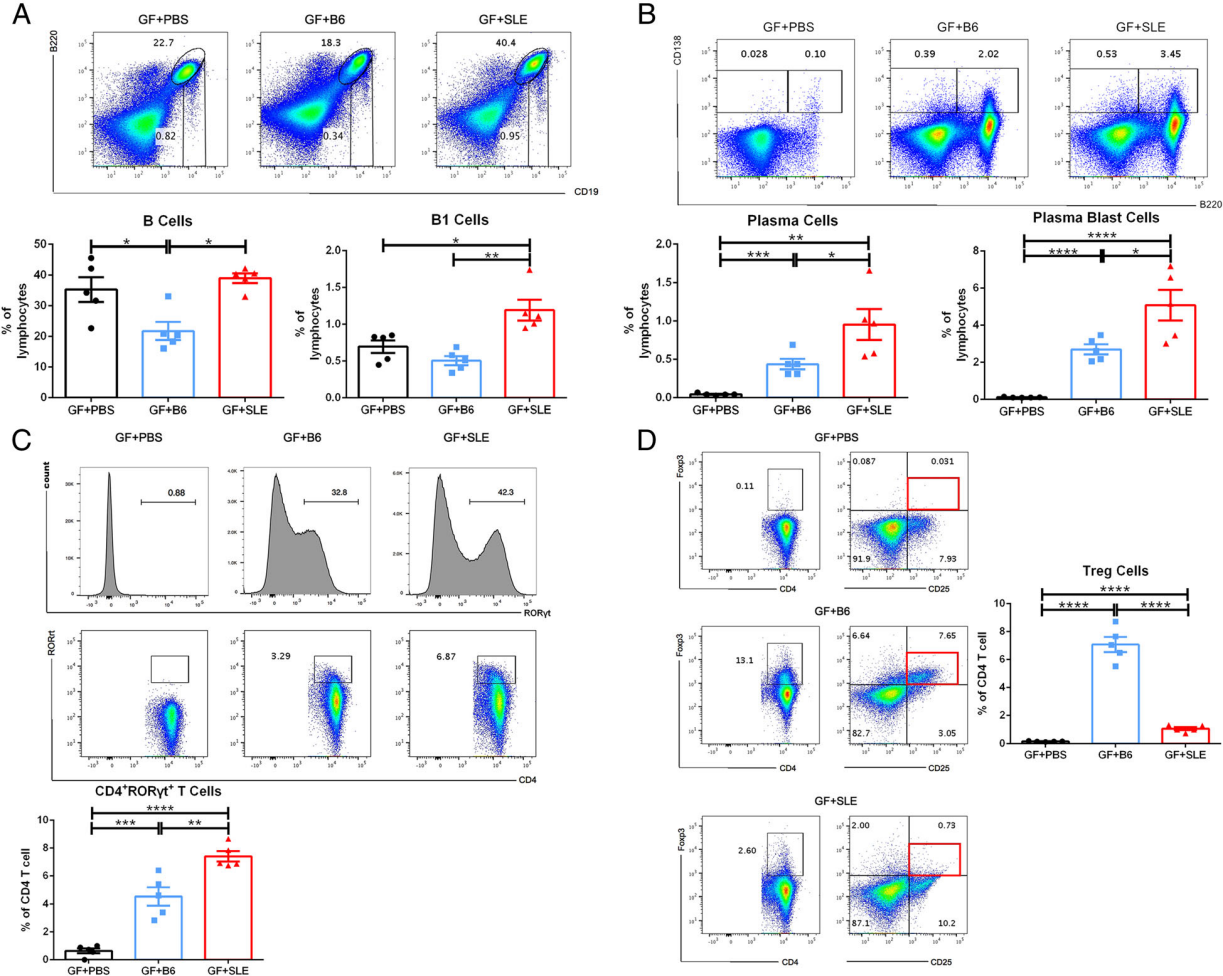
Flow cytometry analysis of the distribution of lymphocytes in the spleens of GF + PBS mice, GF + B6 mice and GF + SLE mice. (A) Plots of B cells and B1 cells (top) and percentages of B cells and B1 cells (5 mice per group, bottom). (B) Plots of plasma cells (PCs) and plasma blast cells (PBs; top) and percentages of PCs and PBs (5 mice per group; bottom). (C) Histograms of RORγt^+^ cells to identify the gate strategy. The number inside the histogram is the percentage of RORγt^+^ cells as of live cells (top); Plots of CD4^+^RORγt^+^T cells gated on CD3e^+^CD4^+^ T cells (middle); and percentages of CD4^+^RORrt^+^ T cells (5 mice per group). (D) Plots regulatory T cells (Tregs, left) and percentages of Tregs in 3 groups (5 mice per group; right). Data represent one of three independent experiments. A Mann-Whitney *t*-test was used to compare groups, and error bars represent means ± SEMs. *P < 0.05, **P < 0.01, ***P < 0.001, ****P < 0.0001

We also examined the status of the peripheral immune system after FMT by use of flow cytometry for measurement of lymphocyte distributions in the spleen. We gated B cells (B220^+^CD19^+^), plasma blast cells (B220^high/medi^CD138^+^), plasma cells (B220^low^CD138^+^), germinal center B cells (GC B cells, CD19^+^CD38^−^CD95^+^PNA^+^) and innate-like B1 cells (CD19^+^B220^med/low^); CD4^+^RORγt^+^ T cells (CD3e^+^CD4^+^RORγt^+^) and regulatory T cells (Treg, CD3e^+^CD4^+^CD25^+^Foxp3^+^). The percentages of B cells and B1 cells were greater in the GF + SLE group than in the GF + B6 group (Fig. 4a). After FMT, there was no change in the distribution of GC B cells in the spleens of recipient mice (data not shown). However, FMT significantly increased the percentages of plasma blast cells (PBs) and plasma cells (PCs) in the GF + SLE group as compared with GF + B6 group. And both groups had more PBs and PCs than GF + PBS group (Fig. 4b).

The populations of T cells also changed according to immune status of the mice. In particular, FMT increased the percentages of CD4^+^RORγt^+^ T cells, and this effect was greater in the GF + SLE group (Fig. 4c). In contrast, FMT also changed the distribution of Treg cells, which were less abundant in the GF + SLE group (Fig. 4d). Taken together, these results indicate that FMT can activate the peripheral immune response. In addition, SLE feces induced a stronger inflammatory response than B6 feces, consistent with its effect on the status of the intestinal mucosa.

### Fecal microbiota from SLE mice induce pathological changes of gene expression in the colon of GF mice

**Fig. 5:**
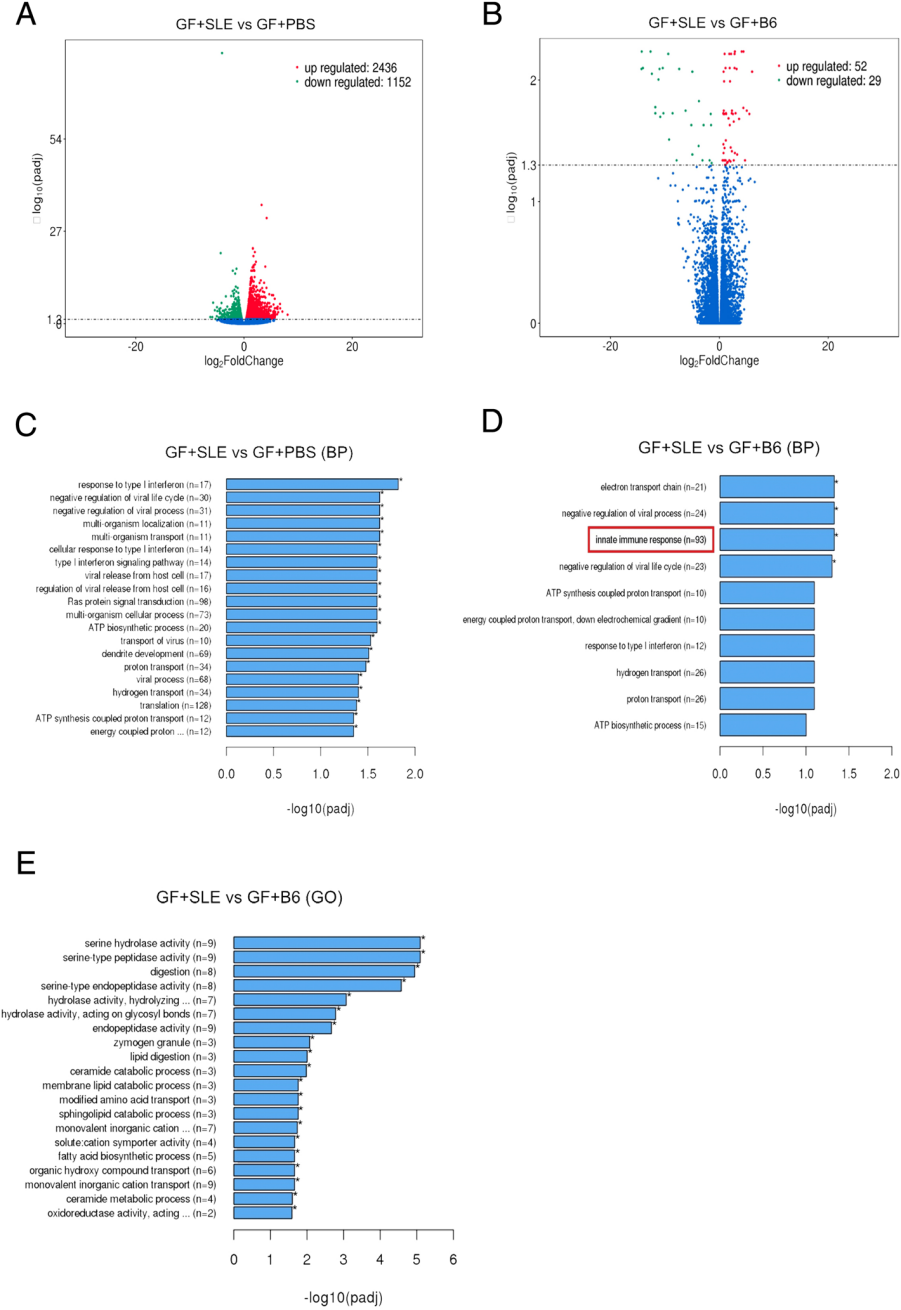
Differential expression of genes (DEGs) in the intestines of GF mice after different treatments. (A) Comparison of the GF + SLE and the GF + PBS groups. (B) Comparison of the GF + SLE and GF + B6 groups. (C) Biological process enrichment of the GF + SLE group relative to the GF + PBS group. (D) Biological process enrichment of the GF + SLE group relative to the GF + B6 group. (E) Gene ortholog (GO) enrichment of the GF + SLE group relative to the GF + B6 group. * beside the bar represents significant difference between groups

We determined the effect of FMT from different mice on the differential expression of genes (DEGs) by analysis of the colon transcriptomes in the different groups. Comparison of GF mice that received SLE feces with GF + PBS mice indicated there were 3588 DEGs (based on a threshold change of 1.3-fold or more, and adjusted for a q-value less than 0.05), with 2436 upregulated genes and 1152 downregulated genes in the GF + SLE group (Fig. 5a). However, comparison of GF + SLE mice with GF + B6 mice indicated there were only 81 DEGs, GF + SLE group had 52 upregulated genes and 29 downregulated genes (Fig. 5b).

We also evaluated the functions of these DEGs. Genes participating in responses to type I interferon were the most significantly enriched in mice that received SLE feces than that received PBS (Fig. 5c). There were 93 DEGs with roles in the innate immune response in GF + SLE mice relative to GF + B6 mice (Fig. 5d). Within the comparison between GF + SLE and GF + B6 groups, there were fewer DEGs, and the most significant difference was in serine hydrolase activity (Fig. 5e). These data demonstrate that FMT of SLE feces triggers alterations of gene expression in the colon that are related to the pathogenesis of SLE. These significant differences indicate that gut microbiota could increase the risk of SLE.

### Fecal microbiota from SLE mice upregulate expression of key SLE susceptibility genes in GF mice

**Fig. 6:**
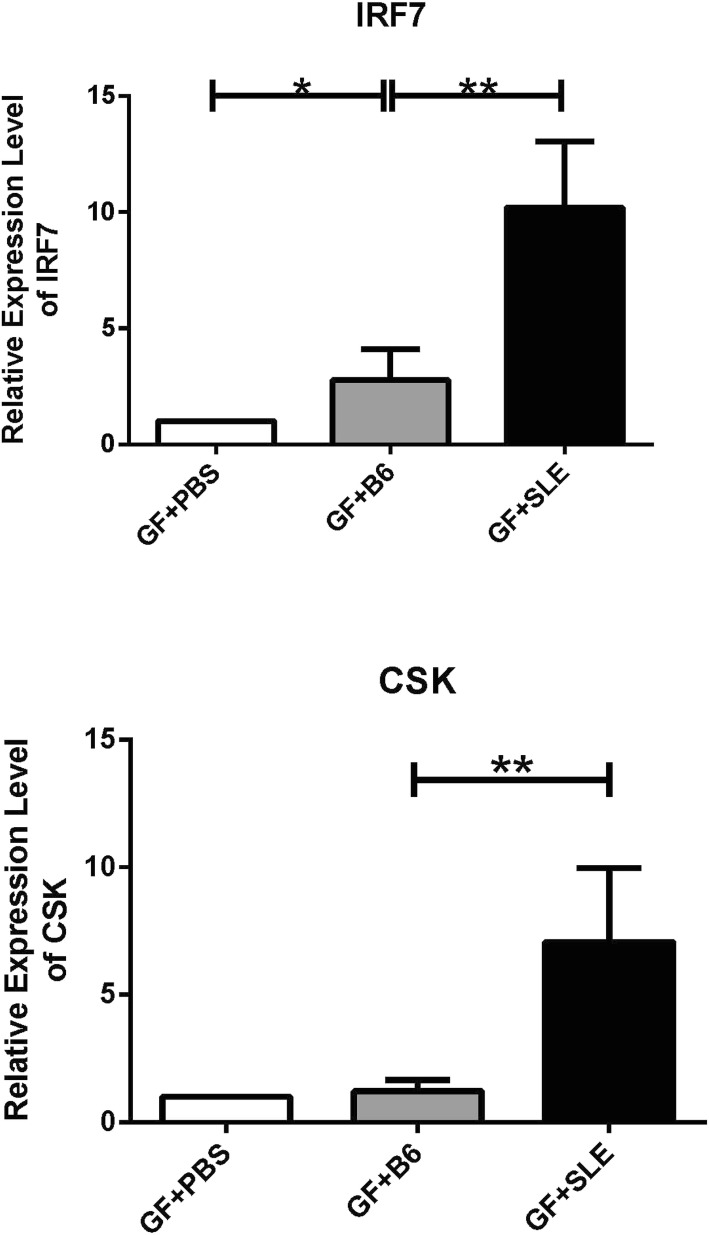
Expression of two major SLE susceptibility genes in GF + PBS mice, GF + B6 mice, and GF + SLE mice. (A) IRF7 (4 mice per group). (B) CSK (4 mice per group). A Mann-Whitney *t*-test was used to compare the two groups. Error bars represent means ± SEMs. *P < 0.05, **P < 0.01

We next used qRT-PCR to measure the expression of *IRF7* and *CSK* (Bentham et al. 2015), the major SLE susceptibility genes in GF + PBS mice, GF + B6 mice, and GF + SLE mice. The expression of *IRF7* (Fig. 6a) and *CSK* (Fig. 6b) genes in GF + SLE group were significantly higher than that in GF + B6 group. The results indicate significantly greater expression of SLE susceptibility genes in GF + SLE mice.

## Discussion

Intestinal microbiota function on the maintenance of health, and abnormal alterations in intestinal microbiota may contribute to the pathogenesis of multiple diseases, although the detailed mechanisms remain unclear. Despite research reported that increased genetic susceptibility to SLE may interact with certain commensal intestinal microbes to trigger chronic autoimmunity in SLE (Greiling et al. 2018), there is only limited evidence showed the microbiota participating in the pathophysiology of SLE. We performed the investigation to study the role of gut microbiota on the pathogenesis of SLE.

We initially compared the gut microbiota of C57/B6 mice with TC (SLE) mice. TC (SLE) mice showed a decreased richness and diversity of microbiota compared with the C57/B6 mice. As previous studies reported in cohorts, patients with SLE showed decreased species richness diversity and taxonomic complexity (Azzouz et al. 2019); the reduced diversity of microbiota was also identified in other inflammatory disease such as IBD (Manichanh et al. 2006). Our study characterized the decreased richness and diversity of the microbiota in TC mice, which was similar to cohorts results. Alterations of microbiota in other mouse models were different from the tendency in clinical studies and our study. In MRL/Mp-Fas^lpr^ (MRL/lpr) model and NZB/W F1 model, the diversity of the microbiota were increased with lupus development (Zhang et al. 2014; Luo et al. 2018). Different results among the murine models may be due to the use of different strains of mice and different sets of control group. In fact, earlier studies reported different results regarding the role of gut microbiota in the development of SLE in mice. In particular, germ-free NZB mice had less severe symptoms as compared with conventional NZB mice (Unni et al. 1975), but there were no clinical differences between germ-free MRL/lpr mice and conventional MRL/lpr mice (Maldonado et al. 1999). Taken together, these results indicate inconsistent results of gut microbiota richness and diversity in SLE among different murine models. There were 5 taxa significantly enriched in the TC mice, including 4 taxa in *Erysipelotrichia* class and 1 taxon in *Actinobacteria* phylum, indicating that the enriched communities were related to SLE. Previous studies also reported an enrichment of gut *Erysipelotrichaceae* in patients with juvenile idiopathic arthritis (JIA) (van Dijkhuizen et al. 2018), and enrichment of unclassified gut *Erysipelotrichaceae* and some classes in *Actinobacteria* phylum were significantly more abundant in patients with multiple sclerosis (MS) (Forbes et al. 2018). These studies suggested that such microbial communities are associate with inflammation-mediated diseases, which were consistent with our results in TC (SLE) mice and supported that some specific microbiota may be involved in the inflammatory process. The mechanism involved needs further studies to identify.

When we transplanted C57/B6 mice feces and TC (SLE) mice feces separately into GF mice. The SourceTracker results identified the gut microbiota of the recipients had great similarity to their donors, and also demonstrated that recipient mice were not precisely the same to donors. Notably, the significantly different species maintained the same differences and trends as donors. Three weeks after FMT, GF mice that received SLE feces had significantly increased serum anti-dsDNA antibodies relative to those that received B6 feces. These results indicate the intestinal microbiota of SLE mice function in the pathogenesis of this disease, and can trigger milder symptoms of SLE in GF mice. However, whether the mono-specie transfer can induce the symptom of disease needs further study.

Lymphocyte distributions in the LP of GF + B6 mice and GF + SLE were different as shown in Fig. 3. The intestinal LP, which underlies the intestinal epithelium, is rich in B and T lymphocytes, and leucocytes in the LP can promote or inhibit inflammatory responses (Abreu 2010). Studies showed that LP CD4^+^ T cells secrete cytokines associated with the development of intestinal inflammation (Kleinschek et al. 2009; Munoz et al. 2009), whereas regulatory T (CD4^+^CD8a^−^CD25^+^Foxp3^+^) cells in the LP can inhibit cytokine production and the development of intestinal inflammation (Makita et al. 2004; Makita et al. 2007). The LP plays the role of an effector, because the lymphocytes there respond to the appropriate stimulation (Brandtzaeg and Pabst 2004). The mucosal immune system in the intestinal tract of GF mice is largely underdeveloped because of an absence of microbiota, so it has fewer lymphocytes than conventional animals (Hooper 2004). We found that administration of intestinal microorganisms to GF mice led to the activation of mucosal immunity and increased lymphocyte. Moreover, there was a significant difference in proportions of lymphocytes in mice that received SLE feces from mice received B6 feces. The GF + SLE group showed an increased CD4^+^RORγt^+^ T cells and decreased Treg cells as compared with GF + B6 group. Previous studies have identified the imbalanced Treg/Th17 cells responses were critical to the pathogenesis of SLE (Rother and van der Vlag 2015), and defective generation of Treg cells can contribute to Th17 responses, proliferation of pro-inflammatory commensals, and subsequent autoimmune responses (Ivanov et al. 2008), indicating that Treg cells may contribute to abnormal Treg/Th17 response. Moreover, LP Th17 cells do not develop in the absence of intestinal microbiota. Although our results showed Th17 cells had similar distributions in mice receiving SLE feces as compared with B6 feces, its direct activator, RORγt, which can regulate the Th17 signature in T cells (Bassolas-Molina et al. 2018; Xiao et al. 2014), was significantly elevated in mice that received SLE feces. In addition, previous research indicated that transplantation of fecal microbiota into GF mice induced Th17 cells at 2 weeks after transfer, and a further increase at 6 weeks after FMT (Ivanov et al. 2008). Our observations which was terminated at 3 weeks after FMT, showed no significant difference in the percentages of Th17 T cells between the groups, probably due to short observation time. However, the CD4^+^RORγt^+^ T cells, which can regulate the Th17 signature in T cells were significantly enriched in the GF + SLE group.

Spleen is an important peripheral lymphoid organ for the development of B cells. Immature B cells pass through transitional stages T1 and T2. A small proportion of transitional B cells form marginal zone B cells; most transitional T2 cells mature into follicular B cells. After antigen encounter, some activated follicular B cells form a germinal center. Short-lived plasma cells are developed from marginal zone B cells and follicular B cells in spleen. Long-lived plasma cells which differentiated from germinal center B cells migrate to bone marrow and reside there for long-term antibody secretion. When self-reactive B cells differentiate into plasma cells, the secreted auto-antibodies, including anti-dsDNA antibodies impair tissue function and induce symptoms of SLE (De Groof et al. 2017; Shapiro-Shelef and Calame 2005). The distribution of lymphocytes in spleen showed the different immune status when introduced with different fecal microbiota. Mice in GF + SLE group had a much more increased percentage of B1 cells. Several lines of research have suggested a role for B1 cells in the pathogenesis of SLE (Berland and Wortis 2002). Plasma blasts, plasma cells and CD4^+^RORγt^+^T cells significantly increased in GF + SLE group, while the Treg cells decreased in GF + SLE group. These results indicated the upregulation of the immune response in the GF + SLE group. Together, distribution of some lymphocytes in both LP and spleen showed a consistent tendency, which also suggest that transfer of gut microbiome from mice with SLE into GF mice led to a more intense immune response as compared with mice transferred with B6 feces.

We used transcriptome sequencing to compare changes in intestinal gene expression after FMT. The comparison of GF + SLE mice with GF + PBS mice showed thousands of differentially expressed genes (DEGs), most of which were related to the innate immune response. The comparison of mice that received SLE feces (GF + SLE) with B6 feces (GF + B6) showed a smaller number of DEGs, and most of the DEGs were related to metabolic pathways and inflammatory immune responses. Mice colonized with SLE feces (GF + SLE) also had significant upregulation of genes related to type I IFN and the innate immune response. Previous studies reported that increased secretion of type I IFN was a characteristic signature of SLE (Crow 2014). The innate immune system initiates inflammation cascades during SLE flares, and continues to fuel adaptive immune responses as the disease progresses (Weidenbusch et al. 2017). These responses also strongly correlated with the onset of SLE. Some of the other genes with expression changed in the present study were associated with virus-related processes as showed in Fig. 5. In fact, other studies reported molecular mimicry between viral antigens and host self-components may trigger SLE. Two studies reported that Epstein-Barr virus (EBV), a dsDNA virus, was active and harbored in patients with SLE (James and Robertson 2012; Ball et al. 2015). In addition, patients with lupus nephritis (LN) often have EBV latent membrane protein 1 and EBV-encoded RNA 1 in biopsy samples (Yu et al. 2014). Thus, there is probably a molecular mimicry between intestinal microbiota and their host, and this could be a risk factor for a lupus flare. In addition, we found far fewer DEGs when compared mice that received SLE feces with B6 feces, and almost all the DEGs in this comparison had roles in metabolism. These data strongly suggest that the different immune status of recipient mice following FMT of SLE feces and B6 feces are associated with the activity of metabolic pathways.

We also used qRT-PCR to analyze the expression of two genes strongly associated with SLE susceptibility. Our results showed significant upregulation of *IRF7* and *CSK* in mice that received SLE feces. Previous studies demonstrated that IFN regulatory factors (IRFs), which are transcription factors downstream of endosomal Toll-like receptors (TLRs) and include *IRF7* (which controls transcription of IFN-I), have pivotal functions in the regulation of innate and adaptive immune responses (Tamura et al. 2008; Xu et al. 2012). Thus, increased *IRF7* expression promotes IFN responses in activated immune cells, and increases the susceptibility to autoimmune diseases (Lee et al. 2014). The protein c-Scr tyrosine kinase (encoded by *CSK*) regulates signal transduction in lymphocytes, and patients with SLE and our mouse model of SLE had upregulation of this gene. More specifically, elevated *CSK* expression is associated with increased risk of SLE and enhanced mature B cell activation and the level of transitional B cells (Manjarrez-Orduno et al. 2012). In addition, our analysis of the DEGs showed that the upregulation of *IRF7* and *CSK* is consistent with functional alterations in the response to type I IFN and the innate immune response. Thus, our results support the interpretation that increased levels of *IRF7* and *CSK* contribute to the onset and/or pathogenesis of SLE.

The present study provided the evidence that there is intestinal microbial dysbiosis in SLE mice with different composition of microbiome from B6 mice. Transplantation of stool samples collected from a mouse model of SLE that had high titer of anti-dsDNA antibodies to GF mice could initiate the lupus-like immune response, including an increased titer of anti-dsDNA antibodies in serum; abnormally increased B cells, GC B cells, CD4^+^RORγt^+^ T cells and decreased Treg cells in lamina propria; and abnormally increased B cells, B1 cells, plasma cells, plasmablasts and decreased Treg cells in spleen. These abnormalities are probably related to the dysregulation of *IRF7* and *CSK* genes which are the key lupus susceptibility genes. Taken together, although the cooperation of both genetic defect and environmental factors can trigger severe SLE, intestinal microbiota alone has the capability to induce some lupus symptoms through its influence on the mucosal and peripheral immune system. This process is probably through the activation of *CSK* gene and *IRF7* gene.

## Conclusion

SLE is associated with alterations of gut microbiota. Fecal microbiota from lupus mice can induce the production of anti-dsDNA antibodies, stimulate the inflammatory immune response, and upregulate the expression of SLE susceptibility genes in recipient germ free mice. Our study indicates that microbiota play an important role in the pathogenesis of SLE. To determine the specific microbial species which are related to the pathogenesis of SLE and the mechanism involved, additional experiments are necessary. Besides, our study provides the potential application that the lupus specific microbial species would be the possible tools for clinical diagnosis of SLE.

## Additional file

Additional file 1: **Table S1.** Alpha diversity indices of donor mice and recipient mice. (DOCX 15 kb)

Additional file 2: **Figure S1.** Gut microbiota composition in donor mice and recipient mice. **Figure S2.** PCA of the microbiota in donor mice and recipient mice. (PDF 295 kb)

Additional file 3: **Figure S3.** Total IgG antibodies in serum of recipient mice. (JPG 134 kb)

## Data Availability

The datasets used in the current study are available from the corresponding author on reasonable request.

## Abbreviations

Anti-dsDNA: Anti-double stranded DNA
B6: C57/B6 mouse
DEGs: Differential expression of genes
EBV: Epstein-Barr virus
ELISA: Enzyme-linked immunosorbent assay
FMT: Fecal microbiota transplantation
GC: Germinal center
GF: Germ free
GO: Gene ontology
IBD: Inflammatory bowel disease
IRFs: IFN regulatory factors
JIA: Juvenile idiopathic arthritis
LN: Lupus nephritis
LP: Lamina propria
MRL/lpr: MRL/Mp-Fas^lpr^ mouse
MS: Multiple sclerosis
NGS: Next-generation sequencing
OTU: Operational taxonomic unit
PBS: Phosphate-buffered saline solution
PBs: Plasma blast cells
PCA: Principal component analysis
PCs: Plasma cells
qRT-PCR: quantitative real-time PCR
RA: Rheumatoid arthritis
SLE: Systemic lupus erythematosus
SPF: Specific pathogen-free
TC: B6.NZM-Sle1^NZM2410/Aeg^Sle2^NZM2410/Aeg^Sle3^NZM2410/Aeg^/LmoJ congenic mouse
TLRs: Toll-like receptors
Treg: Regulatory T cells
