# Supplementary material for: Gut microbiota promote the inflammatory response in the pathogenesis of systemic lupus erythematosus
Source: Mol Med. 2019 Aug 1;25:35. doi: 10.1186/s10020-019-0102-5 (PMC6676588; doi:10.1186/s10020-019-0102-5)
Supplement: Supplementary file 1 — Table S1. Alpha diversity indices of donor mice and recipient mice. (DOCX 15 kb) [file 10020_2019_102_MOESM1_ESM.docx]

**Table S1. Alpha diversity indices of donor mice and recipient mice.**

| **Group** | **Observed_species** | **Chao1** | **ACE** |
| --- | --- | --- | --- |
| C57/B6 | 573 | 683.781 | 677.393 |
| TC (SLE) | 506 | 580.451 | 583.928 |
| GF+B6 | 426 | 453.208 | 457.645 |
| GF+SLE | 421 | 451.961 | 456.183 |
