# Supplementary figures and images for: Gut microbiota promote the inflammatory response in the pathogenesis of systemic lupus erythematosus
Source: Mol Med. 2019 Aug 1;25:35. doi: 10.1186/s10020-019-0102-5 (PMC6676588; doi:10.1186/s10020-019-0102-5)

**A**

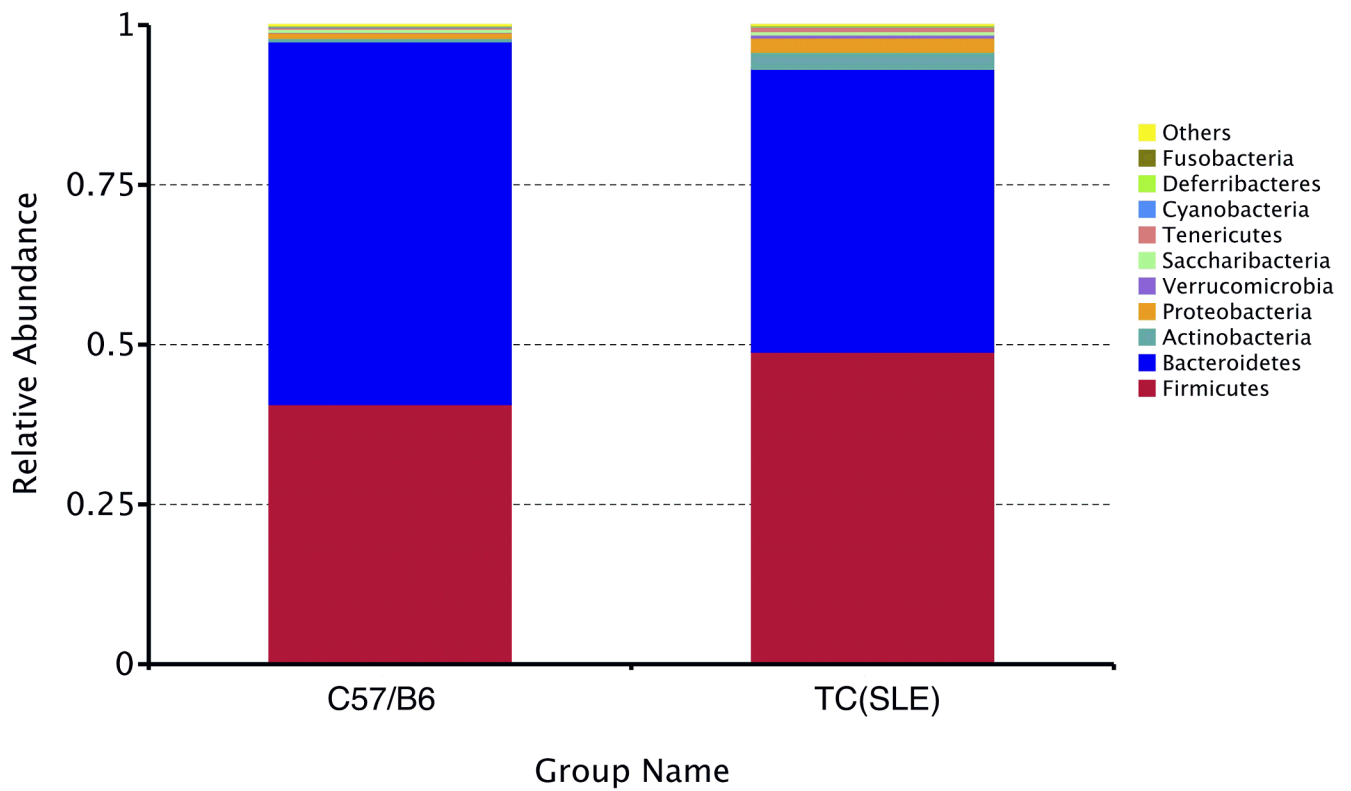

# B

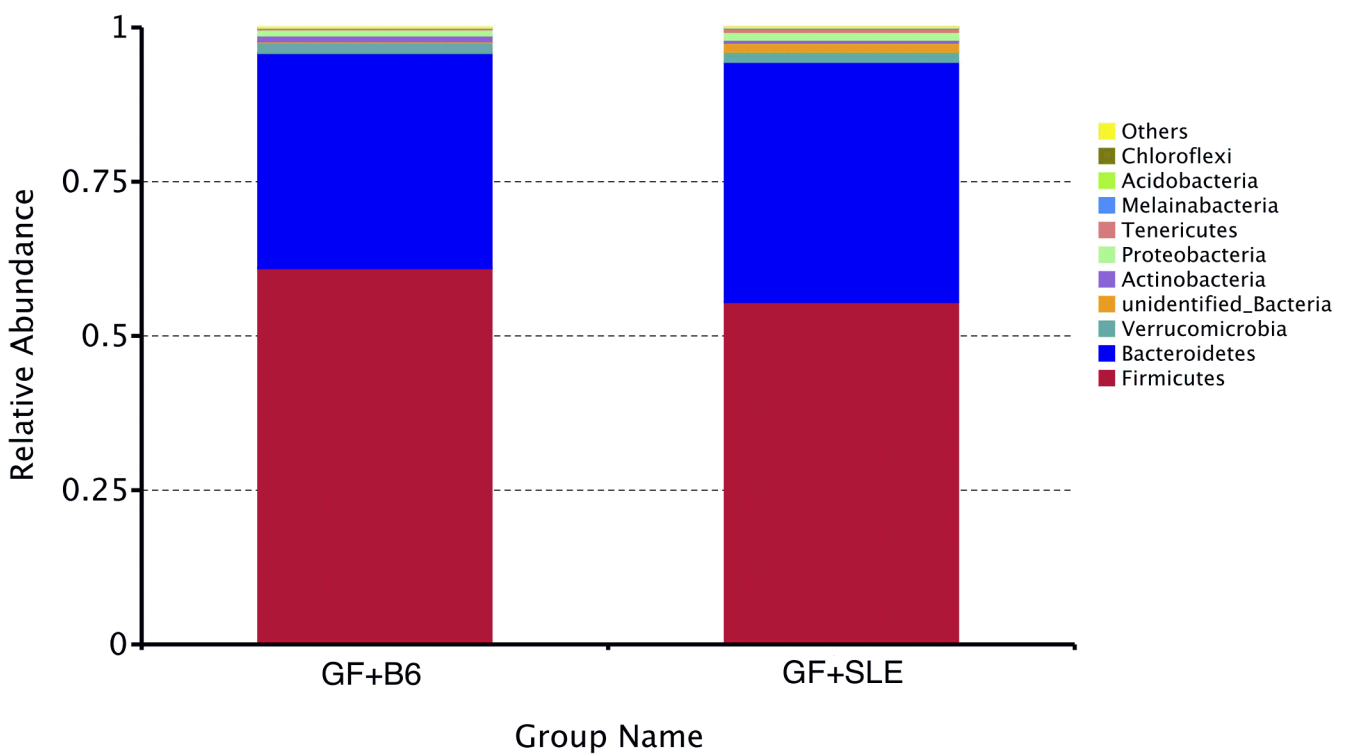

Fig S2

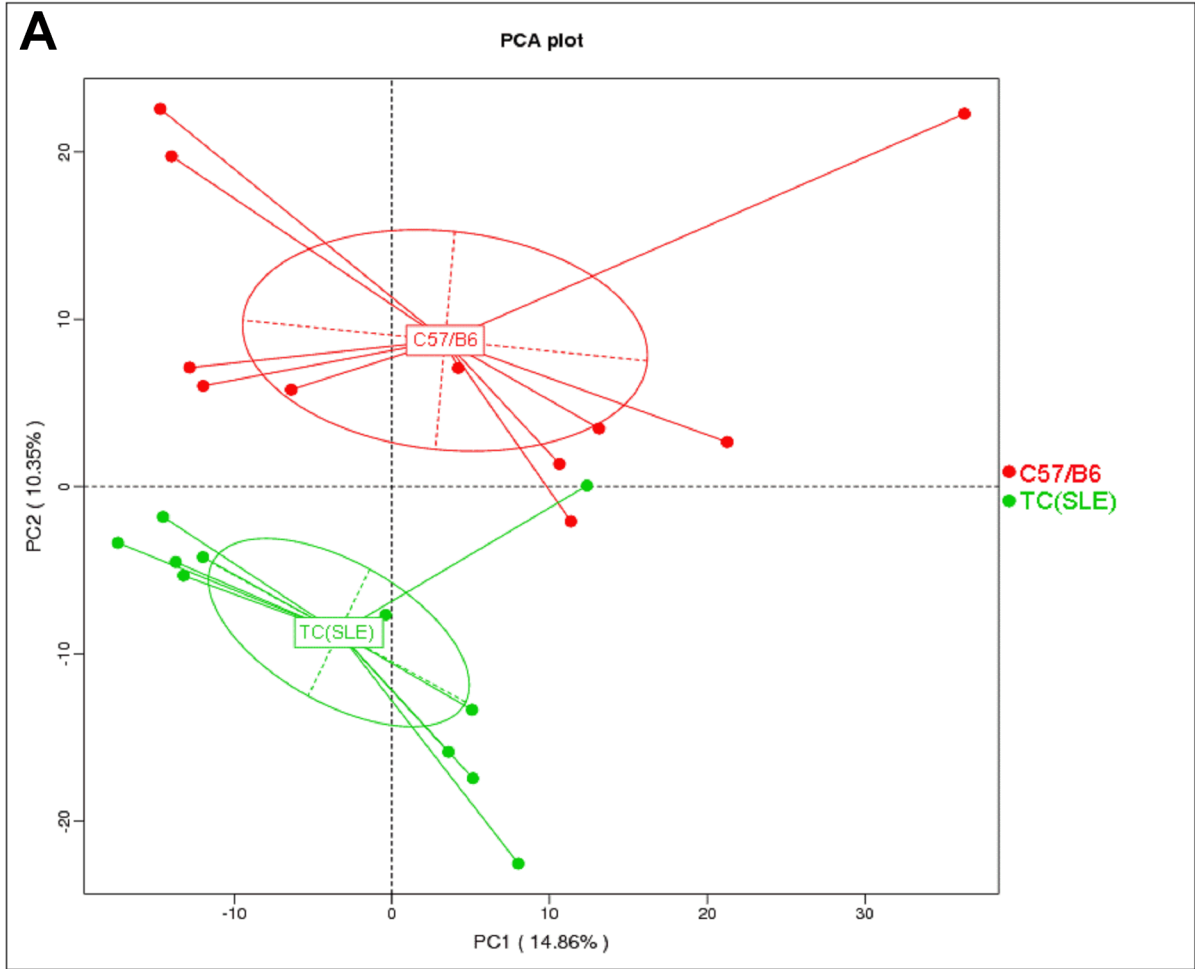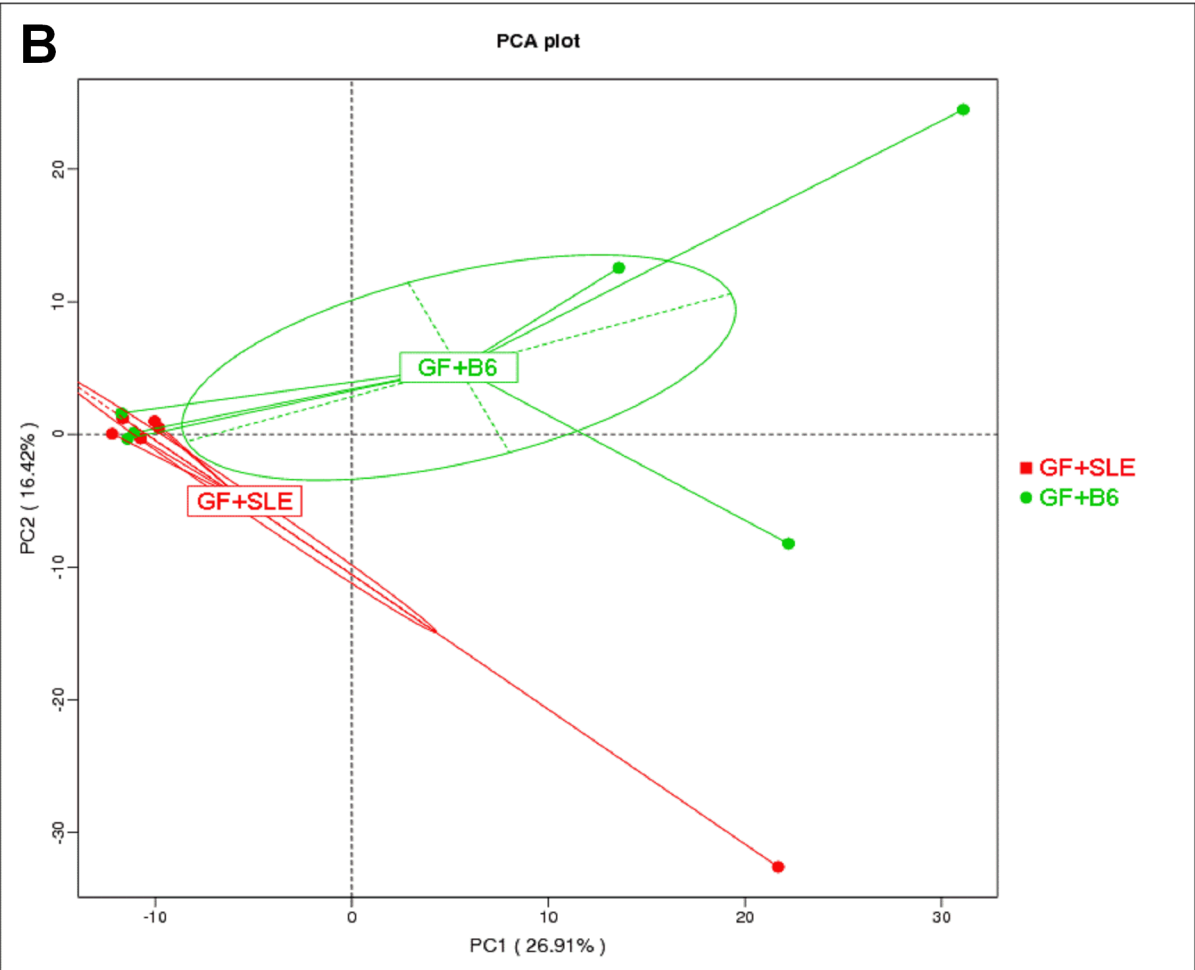

Supplement: Supplementary file 2 — Figure S1. Gut microbiota composition in donor mice and recipient mice. Figure S2. PCA of the microbiota in donor mice and recipient mice. (PDF 295 kb) [file 10020_2019_102_MOESM2_ESM.pdf]

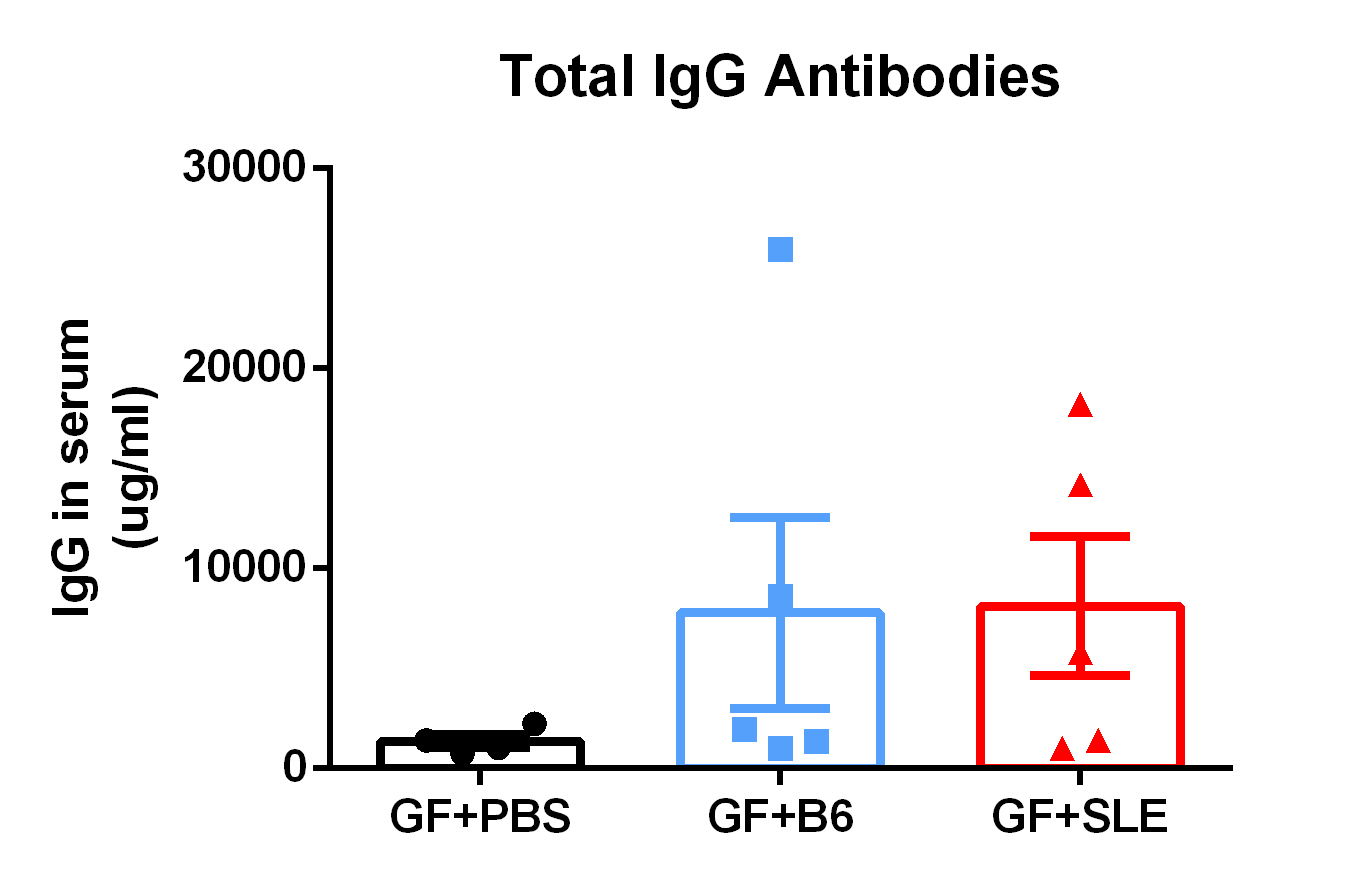

Supplement: Supplementary file 3 — Figure S3. Total IgG antibodies in serum of recipient mice. (JPG 134 kb) [file 10020_2019_102_MOESM3_ESM.jpg]
